# Supplementary material for: Polygenic prediction via Bayesian regression and continuous shrinkage priors
Source: Nat Commun. 2019 Apr 16;10:1776. doi: 10.1038/s41467-019-09718-5 (PMC6467998; doi:10.1038/s41467-019-09718-5)
Supplement: Supplementary file 1 — Supplementary Information [file 41467_2019_9718_MOESM1_ESM.pdf]

Supplementary Information for *Polygenic Prediction via Bayesian Regression and Continuous Shrinkage Priors*

Ge et al.

Supplementary Note

Supplementary Note 1

The Bayesian regression model for PRS-CS and PRS-CS-auto is:

$$\begin{aligned} &\mathbf{y} = \mathbf{Z}\beta + \epsilon, \qquad \epsilon \sim \mathrm{N}(\mathbf{0}, \sigma^2\mathbf{I}), \qquad p(\sigma^2) \propto \sigma^{-2}, \\ &\beta_j \sim \mathrm{N}\left(0, \frac{\sigma^2}{N}\psi_j\right), \qquad \psi_j \sim \mathrm{G}(a, \delta_j), \qquad \delta_j \sim \mathrm{G}(b, \phi), \end{aligned} \tag{1}$$

where $\mathbf{y}$ and $\mathbf{Z}$ have been standardized. The full conditional distributions for all the parameters in this model are analytically tractable, and thus an efficient Gibbs sampler can be derived.

Let $\mathrm{MVN}(\mu, \mathbf{\Sigma})$ denote the multivariate normal distribution with mean μ and covariance $\mathbf{\Sigma}$; $\mathrm{G}(\alpha, \beta)$ and $\mathrm{iG}(\alpha, \beta)$ denote the gamma distribution and inverse-gamma distribution with shape parameter α and scale parameter β, respectively; and $\mathrm{giG}(p, \rho, \chi)$ denote the three-parameter generalized inverse Gaussian distribution with probability density function

$$f(x; \lambda, \rho, \chi) = \frac{(\rho/\chi)^{\lambda/2}}{2K_\lambda(\sqrt{\rho\chi})} x^{\lambda-1} \mathrm{e}^{-(\rho x + \chi/x)/2}, \qquad x > 0, \quad \rho > 0, \quad \chi > 0, \tag{2}$$

where K_λ is the modified Bessel function of the second kind. Let N and M denote the sample size and the total number of genetic markers, respectively. In addition, let $\widehat{\beta} = \mathbf{Z}^\top\mathbf{y}/N$ denote the marginal least squares effect size estimates from the genome-wide association study, $\mathbf{\Psi} = \mathrm{diag}\{\psi_1, \psi_2, \cdots, \psi_M\}$, and $\mathbf{D} = \mathbf{Z}^\top\mathbf{Z}/N$ denote the LD matrix. The Gibbs sampler then involves the following steps in each Markov Chain Monte Carlo (MCMC) iteration:

- update β: $\quad [\, \beta \mid \sigma^2, \mathbf{\Psi}, \widehat{\beta}, \mathbf{D} \,] \sim \mathrm{MVN}(\mu, \mathbf{\Sigma}), \quad \mu = \frac{N}{\sigma^2}\mathbf{\Sigma}\widehat{\beta}, \quad \mathbf{\Sigma} = \frac{\sigma^2}{N}(\mathbf{D} + \mathbf{\Psi}^{-1})^{-1},$
- update σ^2: $\quad [\, \sigma^2 \mid \beta, \mathbf{\Psi}, \widehat{\beta}, \mathbf{D} \,] \sim \mathrm{iG}\left(\frac{N+M}{2}, \frac{N}{2}\left[1 - 2\beta^\top\widehat{\beta} + \beta^\top(\mathbf{D} + \mathbf{\Psi}^{-1})\beta\right]\right),$
- update ψ_j: $\quad [\, \psi_j \mid \beta_j, \sigma^2, \delta_j \,] \sim \mathrm{giG}\left(a - \frac{1}{2}, 2\delta_j, \frac{N\beta_j^2}{\sigma^2}\right),$
- update δ_j: $\quad [\, \delta_j \mid \psi_j \,] \sim \mathrm{G}(a + b, \psi_j + \phi).$

In PRS-CS-auto, we assign a half-Cauchy prior on the global shrinkage parameter[1,2]: $\phi^{1/2} \sim \mathrm{C}^+(0, 1)$, which is equivalent to a scale mixture of gamma distributions: $\phi \sim \mathrm{G}(1/2, \omega)$, $\omega \sim \mathrm{G}(1/2, 1)$. Gibbs updates can then be derived using this augmented representation:

- update ϕ: $\quad [\, \phi \mid \delta_j, \omega \,] \sim \mathrm{G}\left(Mb + \frac{1}{2}, \sum_{j=1}^{M} \delta_j + \omega\right),$

- update ω: $[\,\omega \mid \phi\,] \sim \mathrm{G}(1, 1+\phi)$.

We generate random variates from the generalized inverse Gaussian distribution using an algorithm developed recently[3]. We note that $\mathbf{y}$ and $\mathbf{Z}$ did not appear in any of the updates above, and thus individual-level data is not required for model fitting. In practice, $\mathbf{D}$ and $\boldsymbol{\Psi}$ are $M \times M$ matrices, and the calculation of $(\mathbf{D} + \boldsymbol{\Psi}^{-1})^{-1}$ becomes computationally infeasible when M is large. We thus partition the genome into 1,703 largely independent genomic regions estimated using data from the 1000 Genomes Project European sample[4], and in each MCMC iteration sequentially update the SNP effect sizes within each LD block ℓ:

$$[\,\beta_\ell \mid \sigma^2, \boldsymbol{\Psi}_\ell, \widehat{\beta}_\ell, \mathbf{D}_\ell\,] \sim \mathrm{MVN}(\mu_\ell, \boldsymbol{\Sigma}_\ell), \quad \mu_\ell = \frac{N}{\sigma^2}\boldsymbol{\Sigma}_\ell \widehat{\beta}_\ell, \quad \boldsymbol{\Sigma}_\ell = \frac{\sigma^2}{N}(\mathbf{D}_\ell + \boldsymbol{\Psi}_\ell^{-1})^{-1}. \tag{3}$$

The LD matrix $\mathbf{D}_\ell$ for each LD block can be estimated using an external reference panel.

Supplementary Figures

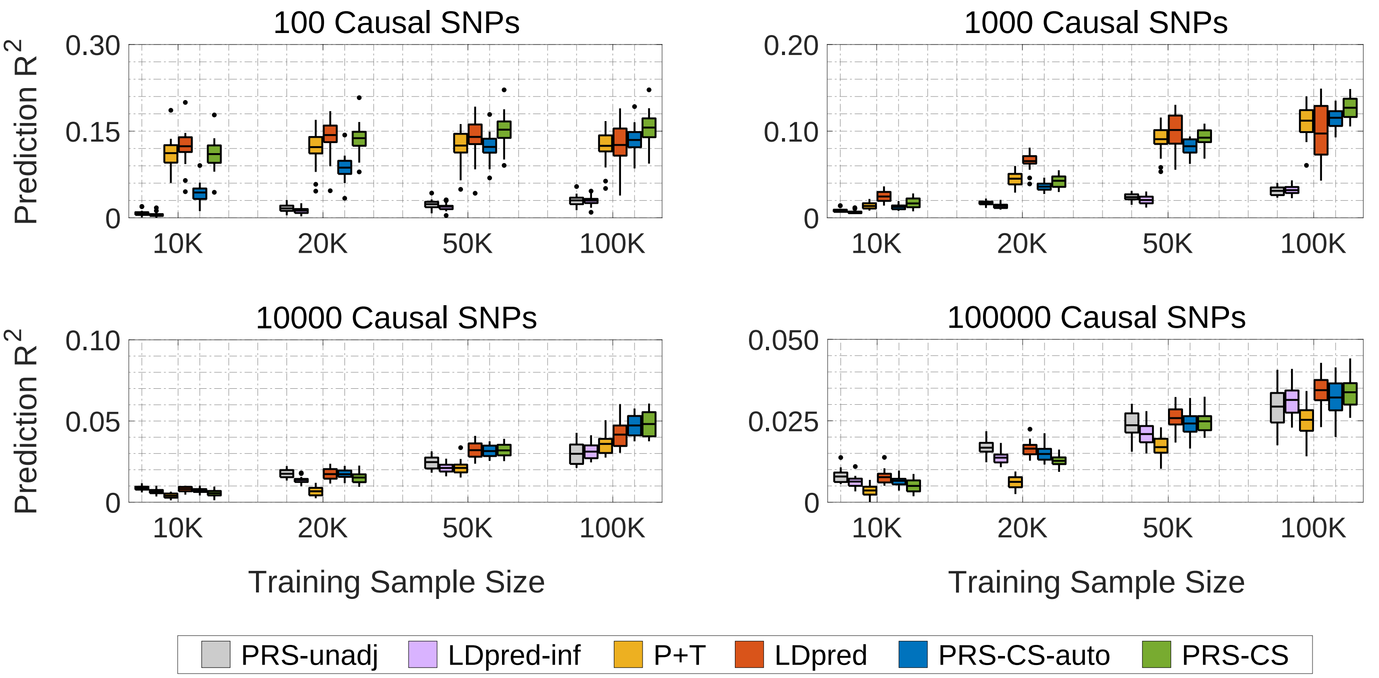

Supplementary Figure 1: Predictive performance of six polygenic prediction methods in simulation studies using a point-normal model with heritability fixed at 0.2. The 1000 Genomes Project European sample was used as an external linkage disequilibrium (LD) reference panel. Tuning parameters (P-value threshold in P+T, fraction of causal variants in LDpred, and global shrinkage parameter in PRS-CS) were selected in a validation data set. Prediction accuracy was quantified by R^2 between the observed and predicted traits in an independent testing set. The four panels correspond to the four genetic architectures (100, 1,000, 10,000 and 100,000 causal variants) simulated using the point-normal model. Within each panel, results for four different training sample sizes (10,000, 20,000, 50,000 and 100,000) are shown. On each box, the central mark is the mean across 20 simulations, the edges of the box are the 25th and 75th percentiles, the whiskers extend to the most extreme data points that are not considered outliers, and the outliers are plotted individually.

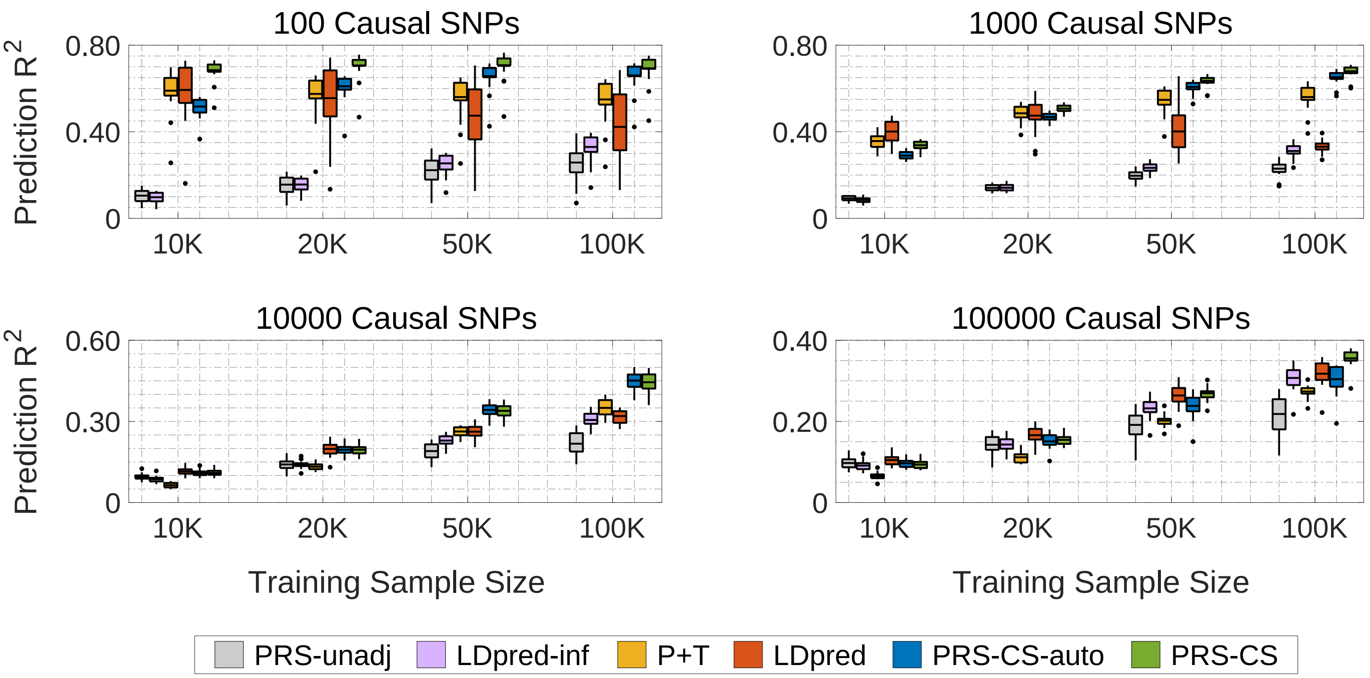

Supplementary Figure 2: Predictive performance of six polygenic prediction methods in simulation studies using a point-normal model with heritability fixed at 0.8. The 1000 Genomes Project European sample was used as an external linkage disequilibrium (LD) reference panel. Tuning parameters (P-value threshold in P+T, fraction of causal variants in LDpred, and global shrinkage parameter in PRS-CS) were selected in a validation data set. Prediction accuracy was quantified by R^2 between the observed and predicted traits in an independent testing set. The four panels correspond to the four genetic architectures (100, 1,000, 10,000 and 100,000 causal variants) simulated using the point-normal model. Within each panel, results for four different training sample sizes (10,000, 20,000, 50,000 and 100,000) are shown. On each box, the central mark is the mean across 20 simulations, the edges of the box are the 25th and 75th percentiles, the whiskers extend to the most extreme data points that are not considered outliers, and the outliers are plotted individually.

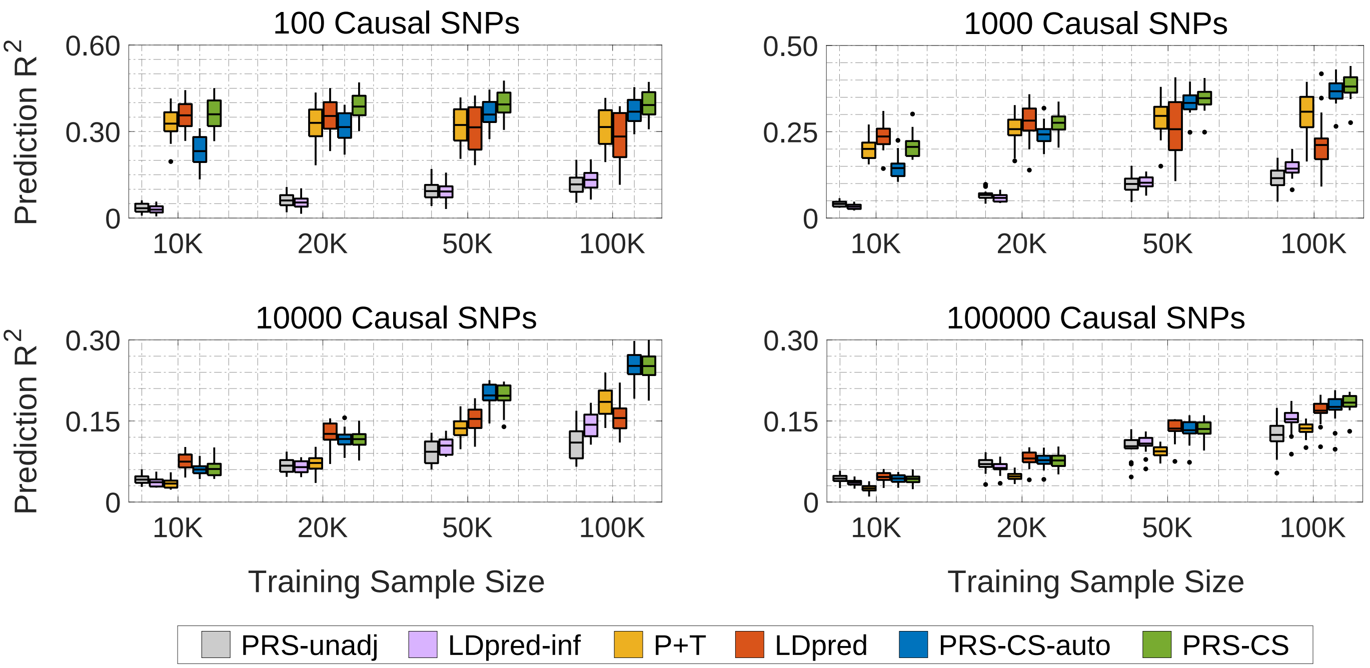

Supplementary Figure 3: Predictive performance of six polygenic prediction methods in simulation studies using a point-t model with heritability fixed at 0.5. The 1000 Genomes Project European sample was used as an external linkage disequilibrium (LD) reference panel. Tuning parameters (P-value threshold in P+T, fraction of causal variants in LDpred, and global shrinkage parameter in PRS-CS) were selected in a validation data set. Prediction accuracy was quantified by R^2 between the observed and predicted traits in an independent testing set. The four panels correspond to the four genetic architectures (100, 1,000, 10,000 and 100,000 causal variants) simulated using the point-t model (a mixture of a point mass at zero and a Student's t-distribution with 4 degrees of freedom). Within each panel, results for four different training sample sizes (10,000, 20,000, 50,000 and 100,000) are shown. On each box, the central mark is the mean across 20 simulations, the edges of the box are the 25th and 75th percentiles, the whiskers extend to the most extreme data points that are not considered outliers, and the outliers are plotted individually.

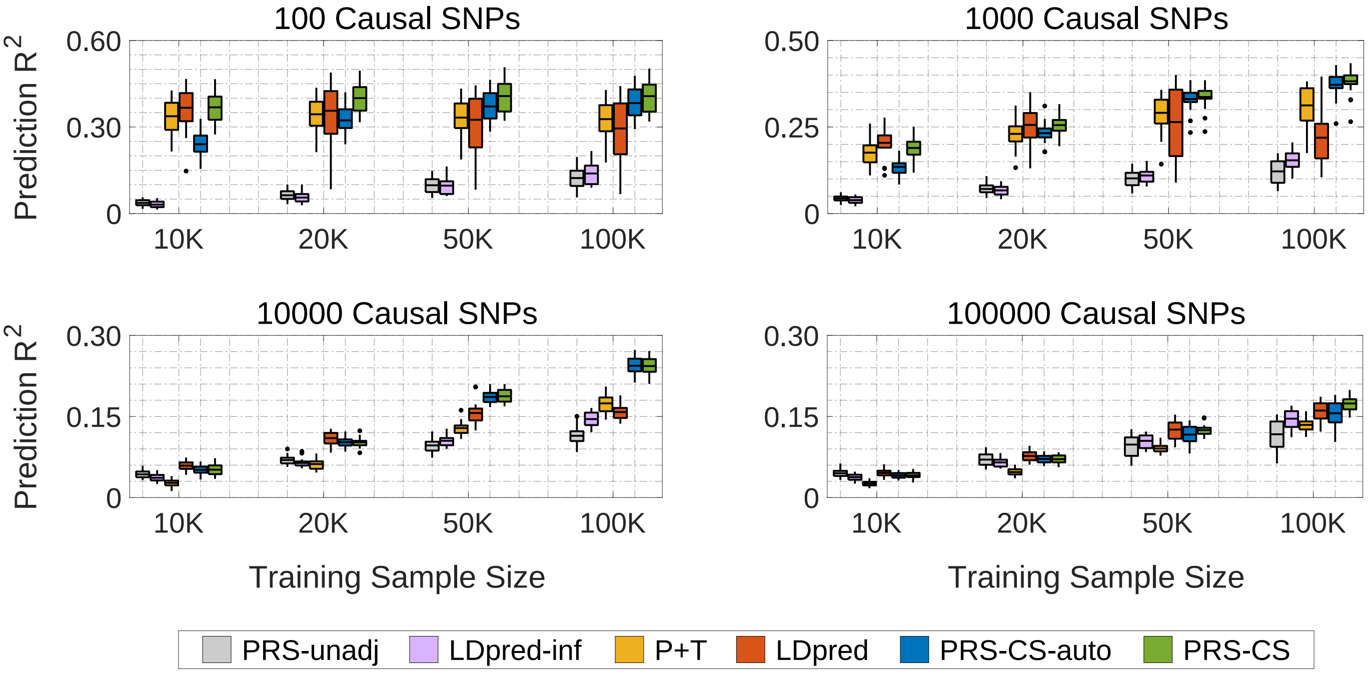

Supplementary Figure 4: Predictive performance of six polygenic prediction methods in simulation studies using a point-gamma model with heritability fixed at 0.5. The 1000 Genomes Project European sample was used as an external linkage disequilibrium (LD) reference panel. Tuning parameters (P-value threshold in P+T, fraction of causal variants in LDpred, and global shrinkage parameter in PRS-CS) were selected in a validation data set. Prediction accuracy was quantified by R^2 between the observed and predicted traits in an independent testing set. The four panels correspond to the four genetic architectures (100, 1,000, 10,000 and 100,000 causal variants) simulated using the point-gamma model (a mixture of a point mass at zero and a gamma distribution with the shape parameter set to 2). Within each panel, results for four different training sample sizes (10,000, 20,000, 50,000 and 100,000) are shown. On each box, the central mark is the mean across 20 simulations, the edges of the box are the 25th and 75th percentiles, the whiskers extend to the most extreme data points that are not considered outliers, and the outliers are plotted individually.

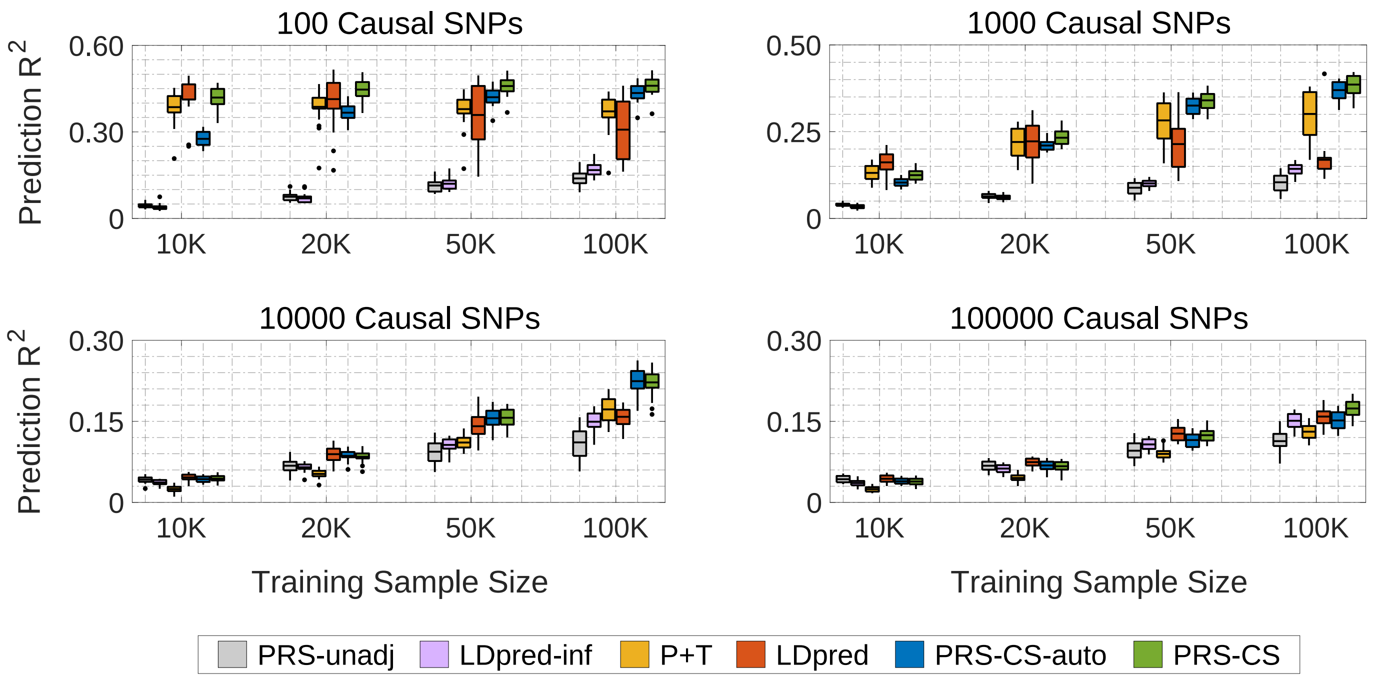

Supplementary Figure 5: Predictive performance of six polygenic prediction methods in simulation studies using an in-sample reference panel. Marker effect sizes were simulated using a point-normal model with different numbers of causal variants and heritability was fixed at 0.5. The combined validation and testing data sets ($N = 6,000$) were used as an in-sample linkage disequilibrium (LD) reference panel. Tuning parameters (P-value threshold in P+T, fraction of causal variants in LDpred, and global shrinkage parameter in PRS-CS) were selected in a validation data set. Prediction accuracy was quantified by R^2 between the observed and predicted traits in an independent testing set. The four panels correspond to the four genetic architectures (100, 1,000, 10,000 and 100,000 causal variants) simulated using the point-normal model. Within each panel, results for four different training sample sizes (10,000, 20,000, 50,000 and 100,000) are shown. On each box, the central mark is the mean across 20 simulations, the edges of the box are the 25th and 75th percentiles, the whiskers extend to the most extreme data points that are not considered outliers, and the outliers are plotted individually.

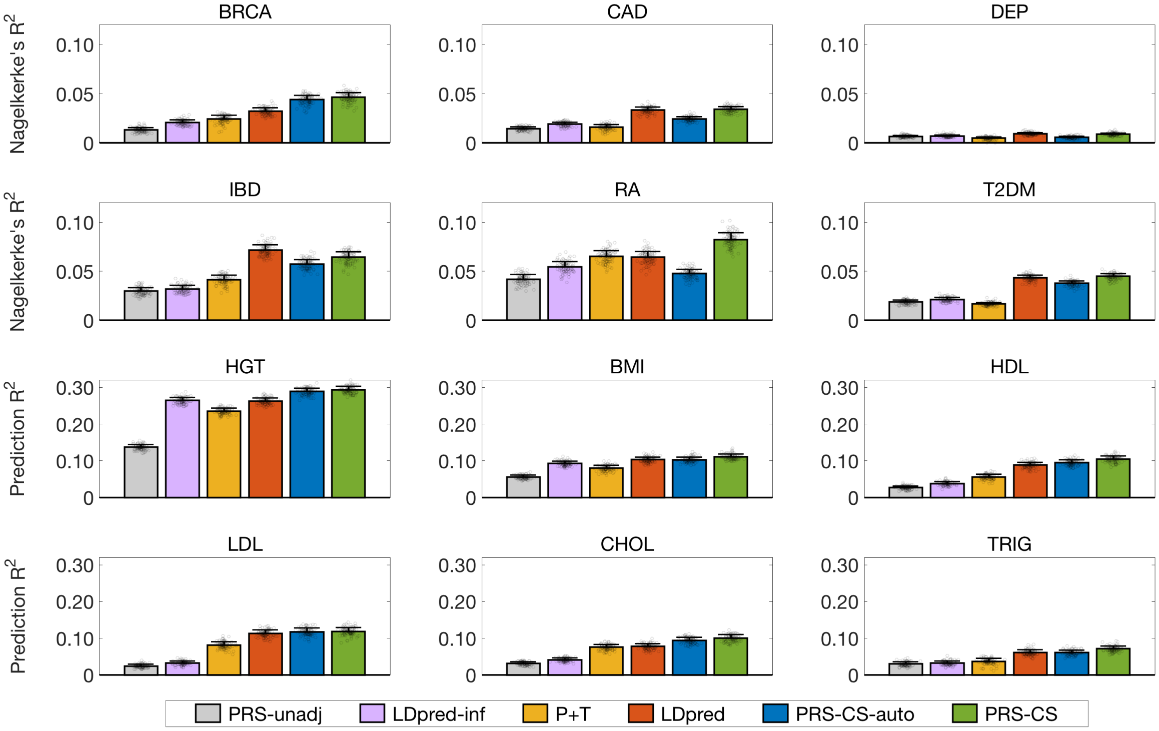

Supplementary Figure 6: Prediction accuracy of six polygenic prediction methods in the Partners HealthCare Biobank using an in-sample reference panel. Posterior effect sizes of single nucleotide polymorphisms (SNPs) were trained with large-scale genome-wide association summary statistics, using the Partners HealthCare Biobank data ($N = 19,136$) as an in-sample linkage disequilibrium (LD) reference panel. Polygenic scores were applied to predict six curated common complex diseases — breast cancer (BRCA), coronary artery disease (CAD), depression (DEP), inflammatory bowel disease (IBD), rheumatoid arthritis (RA), and type 2 diabetes mellitus (T2DM), and six quantitative traits — height (HGT), body mass index (BMI), high-density lipoproteins (HDL), low-density lipoproteins (LDL), cholesterol (CHOL), and triglycerides (TRIG). The Partners HealthCare Biobank sample for each disease and quantitative phenotype was repeatedly and randomly split into a validation set comprising 1/3 of the data and a testing set comprising 2/3 of the data. Tuning parameters (P-value threshold in P+T, fraction of causal SNPs in LDpred, and global shrinkage parameter in PRS-CS) were selected in the validation data set, and the predictive performance was assessed in the testing set. For disease (case-control) phenotypes and quantitive traits, prediction accuracy was measured by the Nagelkerke's R^2 and R^2, respectively, averaged across 100 random splits. The error bar indicates the standard deviation of prediction accuracy across 100 random splits. Prediction accuracy for each random split is overlaid on the bar plot (black circles).

Supplementary Tables

Supplementary Table 1: Numerical results of the simulation studies shown in Fig. 1. SNP effect sizes were simulated using a point-normal model with different numbers of causal variants or a normal mixture model. Heritability was fixed at 0.5. The 1000 Genomes Project European sample was used as an external linkage disequilibrium (LD) reference panel. For each combination of the genetic architecture and the training sample size, the mean and standard deviation of the prediction accuracy for each polygenic prediction method in the testing data set across 20 simulations are reported.

		100 Causal SNPs		1000 Causal SNPs		10000 Causal SNPs		100000 Causal SNPs		Normal Mixture	
		Mean	SD	Mean	SD	Mean	SD	Mean	SD	Mean	SD
10000 subj	PRS-CS	0.4148	0.0326	0.1270	0.0189	0.0418	0.0072	0.0380	0.0072	0.0627	0.0138
	PRS-CS-auto	0.2725	0.0229	0.1008	0.0110	0.0434	0.0062	0.0397	0.0072	0.0555	0.0100
	Ldpred	0.4048	0.0643	0.1583	0.0286	0.0459	0.0071	0.0439	0.0068	0.0688	0.0147
	P+T	0.3815	0.0562	0.1292	0.0238	0.0251	0.0057	0.0242	0.0053	0.0376	0.0137
	Ldpred-inf	0.0390	0.0110	0.0338	0.0064	0.0366	0.0049	0.0358	0.0061	0.0333	0.0070
	PRS-unadj	0.0445	0.0097	0.0397	0.0056	0.0421	0.0061	0.0429	0.0064	0.0397	0.0078
20000 subj	PRS-CS	0.4401	0.0310	0.2260	0.0280	0.0839	0.0121	0.0648	0.0080	0.1030	0.0129
	PRS-CS-auto	0.3606	0.0258	0.2035	0.0192	0.0829	0.0120	0.0663	0.0084	0.1021	0.0125
	Ldpred	0.3930	0.0857	0.2203	0.0626	0.0861	0.0158	0.0735	0.0084	0.1082	0.0161
	P+T	0.3826	0.0621	0.2160	0.0498	0.0522	0.0082	0.0457	0.0071	0.0534	0.0101
	Ldpred-inf	0.0697	0.0161	0.0596	0.0076	0.0642	0.0080	0.0621	0.0077	0.0603	0.0098
	PRS-unadj	0.0751	0.0143	0.0637	0.0092	0.0679	0.0123	0.0678	0.0092	0.0653	0.0095
50000 subj	PRS-CS	0.4496	0.0312	0.3302	0.0377	0.1529	0.0216	0.1231	0.0113	0.1742	0.0175
	PRS-CS-auto	0.4109	0.0303	0.3114	0.0327	0.1509	0.0246	0.1127	0.0164	0.1731	0.0170
	Ldpred	0.3453	0.0937	0.2141	0.0818	0.1385	0.0294	0.1234	0.0144	0.1542	0.0186
	P+T	0.3748	0.0635	0.2725	0.0714	0.1087	0.0153	0.0896	0.0103	0.1195	0.0157
	Ldpred-inf	0.1199	0.0197	0.0980	0.0136	0.1040	0.0150	0.1053	0.0118	0.1029	0.0124
	PRS-unadj	0.1141	0.0229	0.0884	0.0203	0.0938	0.0219	0.0956	0.0167	0.0965	0.0128
100000 subj	PRS-CS	0.4474	0.0325	0.3721	0.0434	0.2153	0.0322	0.1697	0.0160	0.2291	0.0178
	PRS-CS-auto	0.4195	0.0308	0.3543	0.0397	0.2153	0.0363	0.1459	0.0205	0.2311	0.0176
	Ldpred	0.3042	0.0942	0.1793	0.0668	0.1560	0.0253	0.1597	0.0185	0.1616	0.0170
	P+T	0.3664	0.0645	0.2879	0.0774	0.1679	0.0259	0.1316	0.0130	0.1779	0.0170
	Ldpred-inf	0.1659	0.0226	0.1366	0.0208	0.1440	0.0240	0.1463	0.0166	0.1466	0.0150
	PRS-unadj	0.1386	0.0290	0.1042	0.0278	0.1110	0.0299	0.1133	0.0211	0.1181	0.0162

Supplementary Table 2: Numerical results of the simulation studies shown in Supplementary Fig. 1. SNP effect sizes were simulated using a point-normal model with different numbers of causal variants. Heritability was fixed at 0.2. The 1000 Genomes Project European sample was used as an external linkage disequilibrium (LD) reference panel. For each combination of the genetic architecture and the training sample size, the mean and standard deviation of the prediction accuracy for each polygenic prediction method in the testing data set across 20 simulations are reported.

		100 Causal SNPs		1000 Causal SNPs		10000 Causal SNPs		100000 Causal SNPs	
		Mean	SD	Mean	SD	Mean	SD	Mean	SD
10000 subj	PRS-CS	0.1103	0.0272	0.0166	0.0055	0.0055	0.0020	0.0050	0.0020
	PRS-CS-auto	0.0438	0.0169	0.0125	0.0030	0.0072	0.0016	0.0066	0.0016
	Ldpred	0.1239	0.0323	0.0246	0.0063	0.0081	0.0017	0.0077	0.0021
	P+T	0.1120	0.0274	0.0136	0.0042	0.0040	0.0016	0.0036	0.0019
	Ldpred-inf	0.0057	0.0039	0.0068	0.0018	0.0066	0.0015	0.0063	0.0017
	PRS-unadj	0.0078	0.0049	0.0086	0.0022	0.0087	0.0016	0.0079	0.0020
20000 subj	PRS-CS	0.1379	0.0272	0.0427	0.0075	0.0152	0.0034	0.0127	0.0019
	PRS-CS-auto	0.0869	0.0219	0.0360	0.0054	0.0172	0.0029	0.0148	0.0023
	Ldpred	0.1433	0.0319	0.0654	0.0100	0.0173	0.0036	0.0164	0.0024
	P+T	0.1224	0.0313	0.0451	0.0084	0.0067	0.0029	0.0062	0.0020
	Ldpred-inf	0.0125	0.0054	0.0135	0.0030	0.0137	0.0022	0.0137	0.0022
	PRS-unadj	0.0163	0.0064	0.0170	0.0027	0.0175	0.0027	0.0167	0.0025
50000 subj	PRS-CS	0.1526	0.0287	0.0925	0.0111	0.0319	0.0043	0.0248	0.0034
	PRS-CS-auto	0.1227	0.0241	0.0826	0.0093	0.0315	0.0038	0.0242	0.0043
	Ldpred	0.1400	0.0333	0.1014	0.0218	0.0320	0.0051	0.0258	0.0039
	P+T	0.1251	0.0309	0.0907	0.0163	0.0211	0.0044	0.0169	0.0031
	Ldpred-inf	0.0194	0.0069	0.0205	0.0046	0.0211	0.0029	0.0209	0.0037
	PRS-unadj	0.0236	0.0075	0.0239	0.0043	0.0247	0.0042	0.0236	0.0040
100000 subj	PRS-CS	0.1563	0.0283	0.1270	0.0132	0.0481	0.0077	0.0338	0.0048
	PRS-CS-auto	0.1348	0.0245	0.1155	0.0112	0.0473	0.0063	0.0321	0.0058
	Ldpred	0.1261	0.0371	0.0973	0.0329	0.0416	0.0078	0.0344	0.0055
	P+T	0.1246	0.0297	0.1121	0.0196	0.0359	0.0063	0.0253	0.0045
	Ldpred-inf	0.0298	0.0085	0.0319	0.0053	0.0312	0.0048	0.0314	0.0052
	PRS-unadj	0.0302	0.0092	0.0311	0.0051	0.0298	0.0067	0.0294	0.0060

Supplementary Table 3: Numerical results of the simulation studies shown in Supplementary Fig. 2. SNP effect sizes were simulated using a point-normal model with different numbers of causal variants. Heritability was fixed at 0.8. The 1000 Genomes Project European sample was used as an external linkage disequilibrium (LD) reference panel. For each combination of the genetic architecture and the training sample size, the mean and standard deviation of the prediction accuracy for each polygenic prediction method in the testing data set across 20 simulations are reported.

		100 Causal SNPs		1000 Causal SNPs		10000 Causal SNPs		100000 Causal SNPs	
		Mean	SD	Mean	SD	Mean	SD	Mean	SD
10000 subj	PRS-CS	0.6846	0.0492	0.3378	0.0217	0.1095	0.0117	0.0938	0.0105
	PRS-CS-auto	0.5164	0.0463	0.2899	0.0191	0.1088	0.0115	0.0968	0.0112
	Ldpred	0.5935	0.1366	0.4011	0.0547	0.1149	0.0129	0.1047	0.0137
	P+T	0.5899	0.0967	0.3572	0.0352	0.0642	0.0100	0.0646	0.0084
	Ldpred-inf	0.0977	0.0254	0.0849	0.0135	0.0860	0.0111	0.0911	0.0123
	PRS-unadj	0.1050	0.0299	0.0924	0.0121	0.0944	0.0125	0.0974	0.0139
20000 subj	PRS-CS	0.7046	0.0625	0.5069	0.0184	0.1956	0.0168	0.1546	0.0127
	PRS-CS-auto	0.6101	0.0615	0.4680	0.0195	0.1955	0.0174	0.1508	0.0181
	Ldpred	0.5554	0.1663	0.4745	0.0784	0.1982	0.0267	0.1660	0.0190
	P+T	0.5750	0.1013	0.4855	0.0408	0.1328	0.0127	0.1116	0.0128
	Ldpred-inf	0.1564	0.0347	0.1413	0.0172	0.1402	0.0132	0.1430	0.0171
	PRS-unadj	0.1558	0.0437	0.1404	0.0155	0.1408	0.0193	0.1425	0.0225
50000 subj	PRS-CS	0.7048	0.0650	0.6352	0.0266	0.3391	0.0272	0.2694	0.0171
	PRS-CS-auto	0.6571	0.0656	0.6072	0.0270	0.3423	0.0271	0.2383	0.0302
	Ldpred	0.4745	0.1680	0.4015	0.1042	0.2621	0.0229	0.2639	0.0291
	P+T	0.5604	0.1005	0.5476	0.0553	0.2627	0.0196	0.2019	0.0152
	Ldpred-inf	0.2541	0.0503	0.2333	0.0233	0.2292	0.0196	0.2322	0.0247
	PRS-unadj	0.2226	0.0638	0.1968	0.0244	0.1903	0.0308	0.1914	0.0366
100000 subj	PRS-CS	0.6933	0.0690	0.6795	0.0285	0.4449	0.0387	0.3555	0.0213
	PRS-CS-auto	0.6604	0.0694	0.6524	0.0310	0.4512	0.0353	0.3042	0.0361
	Ldpred	0.4226	0.1652	0.3311	0.0297	0.3198	0.0263	0.3176	0.0310
	P+T	0.5494	0.1037	0.5604	0.0595	0.3500	0.0333	0.2737	0.0151
	Ldpred-inf	0.3299	0.0657	0.3111	0.0308	0.3052	0.0299	0.3072	0.0296
	PRS-unadj	0.2579	0.0785	0.2296	0.0337	0.2176	0.0420	0.2183	0.0445

Supplementary Table 4: Numerical results of the simulation studies shown in Supplementary Fig. 3. SNP effect sizes were simulated using a point-t model (a mixture of a point mass at zero and a Student's t-distribution with 4 degrees of freedom) with different numbers of causal variants. Heritability was fixed at 0.5. The 1000 Genomes Project European sample was used as an external linkage disequilibrium (LD) reference panel. For each combination of the genetic architecture and the training sample size, the mean and standard deviation of the prediction accuracy for each polygenic prediction method in the testing data set across 20 simulations are reported.

		100 Causal SNPs		1000 Causal SNPs		10000 Causal SNPs		100000 Causal SNPs	
		Mean	SD	Mean	SD	Mean	SD	Mean	SD
10000 subj	PRS-CS	0.3598	0.0540	0.2061	0.0336	0.0611	0.0141	0.0428	0.0088
	PRS-CS-auto	0.2320	0.0507	0.1447	0.0310	0.0602	0.0114	0.0435	0.0082
	Ldpred	0.3561	0.0523	0.2364	0.0381	0.0748	0.0153	0.0463	0.0094
	P+T	0.3274	0.0566	0.2003	0.0304	0.0342	0.0091	0.0254	0.0071
	Ldpred-inf	0.0293	0.0138	0.0328	0.0074	0.0366	0.0082	0.0363	0.0059
	PRS-unadj	0.0341	0.0152	0.0409	0.0091	0.0415	0.0084	0.0432	0.0082
20000 subj	PRS-CS	0.3866	0.0475	0.2761	0.0320	0.1164	0.0177	0.0769	0.0132
	PRS-CS-auto	0.3155	0.0500	0.2423	0.0310	0.1166	0.0174	0.0774	0.0134
	Ldpred	0.3539	0.0668	0.2823	0.0559	0.1261	0.0255	0.0806	0.0143
	P+T	0.3300	0.0618	0.2576	0.0411	0.0722	0.0153	0.0474	0.0071
	Ldpred-inf	0.0540	0.0215	0.0584	0.0113	0.0645	0.0114	0.0629	0.0108
	PRS-unadj	0.0610	0.0230	0.0669	0.0143	0.0672	0.0134	0.0702	0.0133
50000 subj	PRS-CS	0.3940	0.0477	0.3473	0.0359	0.1967	0.0241	0.1351	0.0148
	PRS-CS-auto	0.3590	0.0496	0.3335	0.0339	0.1972	0.0229	0.1328	0.0205
	Ldpred	0.3147	0.0799	0.2574	0.0803	0.1538	0.0290	0.1359	0.0198
	P+T	0.3227	0.0627	0.2960	0.0543	0.1364	0.0213	0.0938	0.0102
	Ldpred-inf	0.0921	0.0327	0.1023	0.0180	0.1044	0.0168	0.1082	0.0162
	PRS-unadj	0.0934	0.0346	0.0988	0.0237	0.0930	0.0217	0.1032	0.0201
100000 subj	PRS-CS	0.3919	0.0468	0.3811	0.0372	0.2515	0.0318	0.1839	0.0163
	PRS-CS-auto	0.3688	0.0479	0.3673	0.0381	0.2519	0.0291	0.1760	0.0253
	Ldpred	0.2828	0.0850	0.2116	0.0743	0.1554	0.0263	0.1691	0.0211
	P+T	0.3156	0.0698	0.3081	0.0572	0.1853	0.0304	0.1363	0.0125
	Ldpred-inf	0.1325	0.0400	0.1432	0.0249	0.1431	0.0240	0.1533	0.0214
	PRS-unadj	0.1168	0.0425	0.1155	0.0296	0.1100	0.0304	0.1242	0.0267

Supplementary Table 5: Numerical results of the simulation studies shown in Supplementary Fig. 4. SNP effect sizes were simulated using a point-gamma model (a mixture of a point mass at zero and a gamma distribution with the shape parameter set to 2) with different numbers of causal variants. Heritability was fixed at 0.5. The 1000 Genomes Project European sample was used as an external linkage disequilibrium (LD) reference panel. For each combination of the genetic architecture and the training sample size, the mean and standard deviation of the prediction accuracy for each polygenic prediction method in the testing data set across 20 simulations are reported.

		100 Causal SNPs		1000 Causal SNPs		10000 Causal SNPs		100000 Causal SNPs	
		Mean	SD	Mean	SD	Mean	SD	Mean	SD
10000 subj	PRS-CS	0.3683	0.0548	0.1895	0.0313	0.0516	0.0102	0.0410	0.0066
	PRS-CS-auto	0.2403	0.0469	0.1349	0.0246	0.0512	0.0083	0.0409	0.0063
	Ldpred	0.3668	0.0772	0.2044	0.0423	0.0591	0.0085	0.0455	0.0072
	P+T	0.3373	0.0643	0.1759	0.0384	0.0272	0.0066	0.0260	0.0049
	Ldpred-inf	0.0310	0.0117	0.0387	0.0101	0.0366	0.0070	0.0377	0.0065
	PRS-unadj	0.0369	0.0111	0.0436	0.0096	0.0429	0.0071	0.0450	0.0078
20000 subj	PRS-CS	0.4002	0.0551	0.2554	0.0308	0.1024	0.0095	0.0710	0.0077
	PRS-CS-auto	0.3230	0.0523	0.2323	0.0314	0.1024	0.0086	0.0715	0.0079
	Ldpred	0.3564	0.1032	0.2561	0.0600	0.1098	0.0125	0.0769	0.0097
	P+T	0.3441	0.0674	0.2302	0.0400	0.0619	0.0100	0.0471	0.0067
	Ldpred-inf	0.0554	0.0202	0.0669	0.0150	0.0640	0.0081	0.0650	0.0084
	PRS-unadj	0.0633	0.0182	0.0710	0.0147	0.0694	0.0080	0.0702	0.0113
50000 subj	PRS-CS	0.4074	0.0574	0.3367	0.0339	0.1873	0.0121	0.1247	0.0102
	PRS-CS-auto	0.3712	0.0550	0.3309	0.0348	0.1861	0.0122	0.1165	0.0174
	Ldpred	0.3252	0.1012	0.2644	0.1006	0.1563	0.0206	0.1257	0.0179
	P+T	0.3327	0.0745	0.2910	0.0553	0.1285	0.0122	0.0907	0.0083
	Ldpred-inf	0.0968	0.0316	0.1100	0.0216	0.1050	0.0105	0.1049	0.0125
	PRS-unadj	0.0981	0.0285	0.1016	0.0244	0.0965	0.0130	0.0980	0.0215
100000 subj	PRS-CS	0.4071	0.0573	0.3821	0.0392	0.2433	0.0162	0.1740	0.0128
	PRS-CS-auto	0.3831	0.0570	0.3719	0.0393	0.2438	0.0157	0.1559	0.0271
	Ldpred	0.2950	0.1091	0.2191	0.0821	0.1581	0.0136	0.1610	0.0211
	P+T	0.3273	0.0747	0.3125	0.0590	0.1742	0.0181	0.1344	0.0118
	Ldpred-inf	0.1389	0.0403	0.1537	0.0294	0.1451	0.0134	0.1460	0.0189
	PRS-unadj	0.1231	0.0371	0.1218	0.0334	0.1143	0.0181	0.1171	0.0289

Supplementary Table 6: Numerical results of the simulation studies shown in Supplementary Fig. 5. SNP effect sizes were simulated using a point-normal model with different numbers of causal variants. Heritability was fixed at 0.5. The combined validation and testing data sets were used as an in-sample linkage disequilibrium (LD) reference panel. For each combination of the genetic architecture and the training sample size, the mean and standard deviation of the prediction accuracy for each polygenic prediction method in the testing data set across 20 simulations are reported.

		100 Causal SNPs		1000 Causal SNPs		10000 Causal SNPs		100000 Causal SNPs	
		Mean	SD	Mean	SD	Mean	SD	Mean	SD
10000 subj	PRS-CS	0.4191	0.0343	0.1247	0.0168	0.0440	0.0065	0.0386	0.0071
	PRS-CS-auto	0.2759	0.0252	0.1033	0.0115	0.0430	0.0056	0.0390	0.0059
	Ldpred	0.4122	0.0735	0.1616	0.0333	0.0462	0.0066	0.0437	0.0069
	P+T	0.3858	0.0565	0.1312	0.0236	0.0247	0.0063	0.0245	0.0051
	Ldpred-inf	0.0390	0.0111	0.0340	0.0066	0.0369	0.0049	0.0359	0.0061
	PRS-unadj	0.0445	0.0097	0.0397	0.0056	0.0421	0.0061	0.0429	0.0064
20000 subj	PRS-CS	0.4467	0.0334	0.2325	0.0231	0.0845	0.0105	0.0669	0.0099
	PRS-CS-auto	0.3665	0.0273	0.2101	0.0161	0.0861	0.0105	0.0684	0.0086
	Ldpred	0.4136	0.0900	0.2221	0.0613	0.0892	0.0145	0.0745	0.0079
	P+T	0.3868	0.0627	0.2202	0.0455	0.0526	0.0084	0.0451	0.0070
	Ldpred-inf	0.0696	0.0164	0.0604	0.0078	0.0650	0.0079	0.0626	0.0078
	PRS-unadj	0.0751	0.0143	0.0637	0.0092	0.0679	0.0123	0.0678	0.0092
50000 subj	PRS-CS	0.4590	0.0329	0.3402	0.0259	0.1566	0.0171	0.1243	0.0125
	PRS-CS-auto	0.4203	0.0312	0.3251	0.0231	0.1557	0.0199	0.1153	0.0134
	Ldpred	0.3587	0.1089	0.2142	0.0810	0.1409	0.0258	0.1272	0.0130
	P+T	0.3790	0.0615	0.2825	0.0638	0.1108	0.0143	0.0897	0.0108
	Ldpred-inf	0.1202	0.0205	0.1009	0.0122	0.1064	0.0130	0.1073	0.0112
	PRS-unadj	0.1141	0.0229	0.0884	0.0203	0.0938	0.0219	0.0956	0.0167
100000 subj	PRS-CS	0.4600	0.0340	0.3857	0.0302	0.2221	0.0261	0.1738	0.0168
	PRS-CS-auto	0.4348	0.0323	0.3697	0.0271	0.2243	0.0257	0.1516	0.0174
	Ldpred	0.3078	0.1073	0.1690	0.0627	0.1583	0.0190	0.1588	0.0183
	P+T	0.3712	0.0626	0.3010	0.0673	0.1721	0.0239	0.1308	0.0134
	Ldpred-inf	0.1676	0.0238	0.1428	0.0176	0.1493	0.0202	0.1510	0.0150
	PRS-unadj	0.1386	0.0290	0.1042	0.0278	0.1110	0.0299	0.1133	0.0211

Supplementary Table 7: Calibration slopes (the slope of regressing the true phenotype onto the polygenic score predictor) for the six polygenic prediction methods evaluated in Fig. 1 and Supplementary Table 1.

		100 Causal SNPs	1000 Causal SNPs	10000 Causal SNPs	100000 Causal SNPs	Normal Mixture
10000 subj	PRS-CS	1.378	1.642	0.418	0.380	1.169
	PRS-CS-auto	0.776	0.598	0.434	0.427	0.485
	Ldpred	0.912	0.950	0.836	0.858	0.939
	P+T	0.719	1.009	13.251	16.260	5.164
	Ldpred-inf	2.197	2.040	2.047	2.044	2.037
	PRS-unadj	0.005	0.004	0.004	0.004	0.004
20000 subj	PRS-CS	1.288	1.597	0.719	0.405	0.833
	PRS-CS-auto	0.934	0.924	0.737	0.703	0.803
	Ldpred	0.852	0.931	0.829	0.847	0.887
	P+T	0.813	1.009	5.611	9.236	3.334
	Ldpred-inf	2.039	1.850	1.844	1.900	1.929
	PRS-unadj	0.008	0.007	0.007	0.007	0.007
50000 subj	PRS-CS	1.156	1.395	1.012	0.409	1.154
	PRS-CS-auto	0.981	1.074	1.054	1.040	1.099
	Ldpred	0.738	0.790	0.792	0.785	0.857
	P+T	0.949	1.064	2.532	4.623	2.040
	Ldpred-inf	1.720	1.480	1.502	1.538	1.606
	PRS-unadj	0.012	0.010	0.010	0.010	0.011
100000 subj	PRS-CS	1.080	1.272	1.113	0.567	1.223
	PRS-CS-auto	0.951	1.057	1.128	1.121	1.158
	Ldpred	0.514	0.602	0.674	0.707	0.727
	P+T	1.054	1.157	1.837	3.019	1.529
	Ldpred-inf	1.436	1.236	1.263	1.302	1.373
	PRS-unadj	0.015	0.011	0.012	0.012	0.013

Supplementary Table 8: Calibration slopes (the slope of regressing the true phenotype onto the polygenic score predictor) for the six polygenic prediction methods evaluated in Supplementary Fig. 1 and Supplementary Table 2.

		100 Causal SNPs	1000 Causal SNPs	10000 Causal SNPs	100000 Causal SNPs
10000 subj	PRS-CS	1.484	1.084	0.256	0.161
	PRS-CS-auto	0.418	0.255	0.199	0.191
	Ldpred	0.929	0.881	0.895	0.891
	P+T	0.743	1.801	31.957	30.211
	Ldpred-inf	2.011	2.279	2.380	2.311
	PRS-unadj	0.002	0.002	0.002	0.002
20000 subj	PRS-CS	1.392	1.726	0.307	0.185
	PRS-CS-auto	0.707	0.570	0.422	0.396
	Ldpred	0.925	0.980	0.889	0.931
	P+T	0.941	1.110	13.590	17.007
	Ldpred-inf	2.156	2.301	2.271	2.286
	PRS-unadj	0.004	0.004	0.004	0.004
50000 subj	PRS-CS	1.244	1.556	0.727	0.292
	PRS-CS-auto	0.896	0.971	0.745	0.680
	Ldpred	0.835	0.970	0.819	0.801
	P+T	1.085	0.822	5.977	9.032
	Ldpred-inf	1.734	1.820	1.756	1.782
	PRS-unadj	0.007	0.007	0.007	0.007
100000 subj	PRS-CS	1.147	1.476	0.926	0.345
	PRS-CS-auto	0.927	1.099	0.938	0.850
	Ldpred	0.745	0.874	0.726	0.687
	P+T	1.195	0.874	2.780	5.671
	Ldpred-inf	1.499	1.554	1.429	1.463
	PRS-unadj	0.009	0.009	0.008	0.008

Supplementary Table 9: Calibration slopes (the slope of regressing the true phenotype onto the polygenic score predictor) for the six polygenic prediction methods evaluated in Supplementary Fig. 2 and Supplementary Table 3.

		100 Causal SNPs	1000 Causal SNPs	10000 Causal SNPs	100000 Causal SNPs
10000 subj	PRS-CS	1.299	1.717	0.682	0.532
	PRS-CS-auto	0.862	0.859	0.640	0.604
	Ldpred	0.893	0.988	0.888	0.855
	P+T	0.841	0.795	8.034	10.510
	Ldpred-inf	1.964	2.076	2.035	2.020
	PRS-unadj	0.006	0.006	0.006	0.006
20000 subj	PRS-CS	1.197	1.617	1.031	0.384
	PRS-CS-auto	0.944	1.111	0.973	0.911
	Ldpred	0.773	0.971	0.891	0.838
	P+T	0.980	0.723	3.175	5.988
	Ldpred-inf	1.766	1.892	1.792	1.785
	PRS-unadj	0.010	0.010	0.009	0.009
50000 subj	PRS-CS	1.079	1.422	1.251	0.540
	PRS-CS-auto	0.938	1.160	1.193	1.164
	Ldpred	0.623	0.774	0.766	0.778
	P+T	1.120	0.750	1.694	3.345
	Ldpred-inf	1.463	1.554	1.439	1.451
	PRS-unadj	0.014	0.014	0.012	0.012
100000 subj	PRS-CS	0.995	1.303	1.205	0.689
	PRS-CS-auto	0.895	1.113	1.177	1.166
	Ldpred	0.432	0.650	0.691	0.683
	P+T	1.229	0.843	1.415	2.414
	Ldpred-inf	1.224	1.304	1.218	1.221
	PRS-unadj	0.016	0.016	0.014	0.014

Supplementary Table 10: Calibration slopes (the slope of regressing the true phenotype onto the polygenic score predictor) for the six polygenic prediction methods evaluated in Supplementary Fig. 3 and Supplementary Table 4.

		100 Causal SNPs	1000 Causal SNPs	10000 Causal SNPs	100000 Causal SNPs
10000 subj	PRS-CS	1.322	1.550	1.050	0.451
	PRS-CS-auto	0.726	0.674	0.501	0.447
	Ldpred	0.896	0.926	0.855	0.879
	P+T	0.771	0.752	2.786	14.182
	Ldpred-inf	2.114	2.058	2.075	2.107
	PRS-unadj	0.004	0.004	0.004	0.005
20000 subj	PRS-CS	1.239	1.474	0.865	0.704
	PRS-CS-auto	0.894	0.949	0.821	0.746
	Ldpred	0.837	0.940	0.889	0.899
	P+T	0.879	0.764	1.788	8.353
	Ldpred-inf	2.042	1.920	1.894	1.943
	PRS-unadj	0.007	0.007	0.007	0.007
50000 subj	PRS-CS	1.110	1.316	1.098	0.868
	PRS-CS-auto	0.944	1.081	1.073	1.089
	Ldpred	0.771	0.834	0.808	0.839
	P+T	0.993	0.905	1.784	4.280
	Ldpred-inf	1.725	1.642	1.513	1.600
	PRS-unadj	0.011	0.011	0.010	0.011
100000 subj	PRS-CS	1.031	1.221	1.139	0.624
	PRS-CS-auto	0.919	1.060	1.102	1.175
	Ldpred	0.544	0.653	0.677	0.735
	P+T	1.119	0.965	1.649	2.720
	Ldpred-inf	1.493	1.368	1.259	1.357
	PRS-unadj	0.015	0.013	0.012	0.013

Supplementary Table 11: Calibration slopes (the slope of regressing the true phenotype onto the polygenic score predictor) for the six polygenic prediction methods evaluated in Supplementary Fig. 4 and Supplementary Table 5.

		100 Causal SNPs	1000 Causal SNPs	10000 Causal SNPs	100000 Causal SNPs
10000 subj	PRS-CS	1.376	1.587	0.567	0.425
	PRS-CS-auto	0.743	0.667	0.475	0.432
	Ldpred	0.849	0.948	0.886	0.884
	P+T	0.784	0.783	11.090	16.260
	Ldpred-inf	2.173	2.173	2.115	2.149
	PRS-unadj	0.004	0.005	0.005	0.005
20000 subj	PRS-CS	1.275	1.512	0.846	0.529
	PRS-CS-auto	0.914	0.955	0.815	0.719
	Ldpred	0.835	0.948	0.898	0.850
	P+T	0.843	0.857	3.315	8.537
	Ldpred-inf	2.033	1.979	1.925	1.920
	PRS-unadj	0.007	0.007	0.007	0.007
50000 subj	PRS-CS	1.138	1.288	1.187	0.525
	PRS-CS-auto	0.960	1.091	1.129	1.032
	Ldpred	0.729	0.796	0.844	0.794
	P+T	1.033	0.979	1.751	4.306
	Ldpred-inf	1.704	1.597	1.564	1.539
	PRS-unadj	0.012	0.011	0.010	0.010
100000 subj	PRS-CS	1.052	1.228	1.242	0.568
	PRS-CS-auto	0.934	1.070	1.170	1.120
	Ldpred	0.539	0.622	0.700	0.703
	P+T	1.141	1.020	1.639	2.904
	Ldpred-inf	1.458	1.332	1.317	1.294
	PRS-unadj	0.015	0.013	0.013	0.013

Supplementary Table 12: Calibration slopes (the slope of regressing the true phenotype onto the polygenic score predictor) for the six polygenic prediction methods evaluated in Supplementary Fig. 5 and Supplementary Table 6.

		100 Causal SNPs	1000 Causal SNPs	10000 Causal SNPs	100000 Causal SNPs
10000 subj	PRS-CS	1.419	1.637	0.456	0.406
	PRS-CS-auto	0.812	0.633	0.453	0.438
	Ldpred	0.943	0.983	0.858	0.871
	P+T	0.705	0.981	13.624	16.006
	Ldpred-inf	2.217	2.077	2.085	2.071
	PRS-unadj	0.005	0.004	0.004	0.004
20000 subj	PRS-CS	1.324	1.653	0.806	0.540
	PRS-CS-auto	0.986	0.980	0.788	0.744
	Ldpred	0.899	0.973	0.865	0.877
	P+T	0.779	0.966	5.634	8.884
	Ldpred-inf	2.063	1.901	1.890	1.940
	PRS-unadj	0.008	0.007	0.007	0.007
50000 subj	PRS-CS	1.220	1.447	1.122	0.447
	PRS-CS-auto	1.057	1.159	1.133	1.111
	Ldpred	0.787	0.841	0.843	0.836
	P+T	0.915	0.978	2.456	4.415
	Ldpred-inf	1.754	1.551	1.561	1.596
	PRS-unadj	0.012	0.010	0.010	0.010
100000 subj	PRS-CS	1.153	1.343	1.213	0.630
	PRS-CS-auto	1.054	1.147	1.232	1.225
	Ldpred	0.604	0.679	0.722	0.732
	P+T	1.012	0.991	1.724	2.864
	Ldpred-inf	1.483	1.323	1.337	1.377
	PRS-unadj	0.015	0.011	0.012	0.012

Supplementary Table 13: SNP heritability of the six common complex diseases (breast cancer, coronary artery disease, depression, inflammatory bowel disease, rheumatoid arthritis, and type 2 diabetes mellitus), and six quantitative traits (height, body mass index, high-density lipoproteins, low-density lipoproteins, cholesterol, and triglycerides), on the observed scale and the liability scale, estimated using genome-wide association summary statistics and LD score regression.

Disease/Trait	Abbreviation	GWAS sample prevalence	Assumed population prevalence	SNP heritability (observed scale)	SNP heritability (liability scale)
Breast Cancer	BRCA	53.71%	12.5%	0.132 (0.012)	0.149 (0.014)
Coronary Artery Disease	CAD	32.99%	5%	0.068 (0.005)	0.065 (0.005)
Depression	DEP	34.59%	15%	0.076 (0.004)	0.100 (0.006)
Inflammatory Bowel Disease	IBD	37.18%	0.5%	0.321 (0.032)	0.163 (0.016)
Rheumatoid Arthritis	RA	24.64%	0.5%	0.142 (0.019)	0.091 (0.012)
Type 2 Diabetes Mellitus	T2DM	16.76%	10%	0.077 (0.006)	0.145 (0.011)
Height	HGT	NA	NA	0.454 (0.020)	NA
Body mass index	BMI	NA	NA	0.192 (0.005)	NA
High-density lipoproteins	HDL	NA	NA	0.093 (0.014)	NA
Low-density lipoproteins	LDL	NA	NA	0.084 (0.014)	NA
Cholesterol	CHOL	NA	NA	0.096 (0.013)	NA
Triglycerides	TRIG	NA	NA	0.094 (0.018)	NA

Supplementary References

1. Gelman, A. Prior distributions for variance parameters in hierarchical models. *Bayesian Analysis* **1**, 515–534 (2006).

2. Polson, N. & Scott, J. Shrink globally, act locally: Sparse bayesian regularization and prediction. *Bayesian Statistics* **9**, 501–538 (2010).

3. Devroye, L. Random variate generation for the generalized inverse Gaussian distribution. *Statistics and Computing* **24**, 239–246 (2014).

4. Berisa, T. & Pickrell, J. Approximately independent linkage disequilibrium blocks in human populations. *Bioinformatics* **32**, 283–285 (2016).
