# Supplementary material for: Polygenic prediction via Bayesian regression and continuous shrinkage priors
Source: Nat Commun. 2019 Apr 16;10:1776. doi: 10.1038/s41467-019-09718-5 (PMC6467998; doi:10.1038/s41467-019-09718-5)
Supplement: Supplementary file 3 — Description of Additional Supplementary Files [file 41467_2019_9718_MOESM3_ESM.pdf]

Description of Additional Supplementary Files

Supplementary Data 1: Information on the genome-wide association summary statistics of the six common complex diseases (breast cancer, coronary artery disease, depression, inflammatory bowel disease, rheumatoid arthritis, and type 2 diabetes mellitus), and six quantitative traits (height, body mass index, high-density lipoproteins, low-density lipoproteins, cholesterol, and triglycerides).

Supplementary Data 2: Numerical values of the prediction accuracy shown in Fig. 2. Polygenic scores were trained with large-scale genome-wide association summary statistics, using the 1000 Genomes Project European sample as an external linkage disequilibrium (LD) reference panel. For each of the curated diseases (breast cancer, coronary artery disease, depression, inflammatory bowel disease, rheumatoid arthritis, and type 2 diabetes mellitus), and quantitative traits (height, body mass index, high-density lipoproteins, low-density lipoproteins, cholesterol, and triglycerides), the Partners HealthCare Biobank sample was repeatedly and randomly split into a validation set comprising 1/3 of the data and a testing set comprising 2/3 of the data. Tuning parameters (*P*-value threshold in P+T, fraction of causal variants in LDpred, and global shrinkage parameter in PRS-CS) were selected in the validation data set, and the predictive performance was assessed in the testing set. The mean and standard deviation of each prediction accuracy metric (R^2 or Nagelkerke's R^2, area under the receiver operating characteristic [ROC] curve, area under the precision-call curve, odds ratio [OR] comparing top 10% of the participants having high polygenic risk with the remaining 90% of the sample) for each polygenic prediction method across 100 random splits are reported.

Supplementary Data 3: Numerical values of the prediction accuracy shown in Supplementary Fig. 6. Polygenic scores were trained with large-scale genome-wide association summary statistics, using the Partners HealthCare Biobank data as an in-sample linkage disequilibrium (LD) reference panel. For each of the curated diseases (breast cancer, coronary artery disease, depression, inflammatory bowel disease, rheumatoid arthritis, and type 2 diabetes mellitus), and quantitative traits (height, body mass index, high-density lipoproteins, low-density lipoproteins, cholesterol, and triglycerides), the Partners HealthCare Biobank sample was repeatedly and randomly split into a validation set comprising 1/3 of the data and a testing set comprising 2/3 of the data. Tuning parameters (P-value threshold in P+T, fraction of causal variants in LDpred, and global shrinkage parameter in PRS-CS) were selected in the validation data set, and the predictive performance was assessed in the testing set. The mean and standard deviation of each prediction accuracy metric (R2 or Nagelkerke's R2, area under the receiver operating characteristic [ROC] curve, area under the precision-call curve, odds ratio [OR] comparing top 10% of the participants having high polygenic risk with the remaining 90% of the sample) for each polygenic prediction method across 100 random splits are reported.
