# Supplementary material for: Polygenic prediction via Bayesian regression and continuous shrinkage priors
Source: Nat Commun. 2019 Apr 16;10:1776. doi: 10.1038/s41467-019-09718-5 (PMC6467998; doi:10.1038/s41467-019-09718-5)
Supplement: Supplementary file 7 — Reporting Summary [file 41467_2019_9718_MOESM7_ESM.pdf]

nature research

Corresponding author(s): Tian Ge

Last updated by author(s): Mar 16, 2019

Reporting Summary

Nature Research wishes to improve the reproducibility of the work that we publish. This form provides structure for consistency and transparency in reporting. For further information on Nature Research policies, see Authors & Referees and the Editorial Policy Checklist.

Statistics

For all statistical analyses, confirm that the following items are present in the figure legend, table legend, main text, or Methods section.

n/a	Confirmed	
☐	☒	The exact sample size (*n*) for each experimental group/condition, given as a discrete number and unit of measurement
☐	☒	A statement on whether measurements were taken from distinct samples or whether the same sample was measured repeatedly
☐	☒	The statistical test(s) used AND whether they are one- or two-sided<br>*Only common tests should be described solely by name; describe more complex techniques in the Methods section.*
☐	☒	A description of all covariates tested
☐	☒	A description of any assumptions or corrections, such as tests of normality and adjustment for multiple comparisons
☐	☒	A full description of the statistical parameters including central tendency (e.g. means) or other basic estimates (e.g. regression coefficient) AND variation (e.g. standard deviation) or associated estimates of uncertainty (e.g. confidence intervals)
☐	☒	For null hypothesis testing, the test statistic (e.g. *F*, *t*, *r*) with confidence intervals, effect sizes, degrees of freedom and *P* value noted<br>*Give P values as exact values whenever suitable.*
☐	☒	For Bayesian analysis, information on the choice of priors and Markov chain Monte Carlo settings
☐	☒	For hierarchical and complex designs, identification of the appropriate level for tests and full reporting of outcomes
☐	☒	Estimates of effect sizes (e.g. Cohen's *d*, Pearson's *r*), indicating how they were calculated

Our web collection on statistics for biologists contains articles on many of the points above.

Software and code

Policy information about availability of computer code

Data collection	No software was used.
Data analysis	PRS-CS: https://github.com/getian107/PRScs<br>Eagle2: https://data.broadinstitute.org/alkesgroup/Eagle<br>Genome partition: http://bitbucket.org/nygcresearch/ldetect-data<br>LDpred: https://github.com/bvilhjal/ldpred<br>Minimac3: https://genome.sph.umich.edu/wiki/Minimac3<br>PLINK 1.9: https://www.cog-genomics.org/plink/1.9<br>PRSice-2: https://choishingwan.github.io/PRSice

For manuscripts utilizing custom algorithms or software that are central to the research but not yet described in published literature, software must be made available to editors/reviewers. We strongly encourage code deposition in a community repository (e.g. GitHub). See the Nature Research guidelines for submitting code & software for further information.

Data

Policy information about availability of data

All manuscripts must include a data availability statement. This statement should provide the following information, where applicable:

- Accession codes, unique identifiers, or web links for publicly available datasets
- A list of figures that have associated raw data
- A description of any restrictions on data availability

UK Biobank data are available to registered investigators under approved applications [http://www.ukbiobank.ac.uk]. All genome-wide association summary statistics used in this study are publicly available. Download links are included in Supplementary Data 1. Other relevant data are available from the corresponding author upon request.

Field-specific reporting

Please select the one below that is the best fit for your research. If you are not sure, read the appropriate sections before making your selection.

☒ Life sciences ☐ Behavioural & social sciences ☐ Ecological, evolutionary & environmental sciences

For a reference copy of the document with all sections, see nature.com/documents/nr-reporting-summary-flat.pdf

Life sciences study design

All studies must disclose on these points even when the disclosure is negative.

Sample size	In simulation studies, we considered a range of different training sample sizes. For real data analyses, we used all the samples collected by the Partners Healthcare Biobank.
Data exclusions	We excluded samples that did not pass the quality control of genetic data. When predicting complex diseases in the Partners Healthcare Biobank, we used all the curated cases and controls. When predicting quantitative traits in the Partners Healthcare Biobank, we used a relatively heathy population with a Charlson age-comorbidity index 0-2 and the predicted 10-year survival probability greater than 90% to avoid measurements affected by severe diseases or medications.
Replication	The new statistical method was tested across a wide range of different simulation settings using data from the UK Biobank, and was tested on six common complex diseases and six quantitative traits in the Partners Healthcare Biobank.
Randomization	Randomization is not relevant in this study and was not employed.
Blinding	Blinding is not relevant in this study and was not employed.

Reporting for specific materials, systems and methods

We require information from authors about some types of materials, experimental systems and methods used in many studies. Here, indicate whether each material, system or method listed is relevant to your study. If you are not sure if a list item applies to your research, read the appropriate section before selecting a response.

Materials & experimental systems

n/a	Involved in the study	
☒	☐	Antibodies
☒	☐	Eukaryotic cell lines
☒	☐	Palaeontology
☒	☐	Animals and other organisms
☐	☒	Human research participants
☒	☐	Clinical data

Methods

n/a	Involved in the study	
☒	☐	ChIP-seq
☒	☐	Flow cytometry
☒	☐	MRI-based neuroimaging

Human research participants

Policy information about studies involving human research participants

Population characteristics	UK Biobank is a prospective cohort study of ~500,000 individuals (age 40–69 years) recruited across Great Britain during 2006–2010. Majority of the participants have been genotyped.<br><br>The Partners Healthcare Biobank has enrolled more than 96,000 participants, and released genome-wide genetic data for 25,482 subjects.
Recruitment	UK Biobank is a prospective cohort study of ~500,000 individuals recruited across Great Britain during 2006-2010.<br><br>The Partners Healthcare Biobank is a collection of plasma, serum, DNA and buffy coats samples collected from consented subjects, which are linked to their electronic health records (EHR) and survey data on lifestyle, environment, and family history.
Ethics oversight	UK Biobank data for the current analyses were obtained under an approved data request to the UK Biobank access team. A study protocol is not required for Partners investigators to obtain de-identified data sets from the Partners HealthCare Biobank.

Note that full information on the approval of the study protocol must also be provided in the manuscript.
